# Dual-Angle Interferometric Scattering Microscopy for Optical Multiparametric Particle Characterization

**DOI:** 10.1021/acs.nanolett.3c03539

**Published:** 2024-01-31

**Authors:** Erik Olsén, Berenice García Rodríguez, Fredrik Skärberg, Petteri Parkkila, Giovanni Volpe, Fredrik Höök, Daniel Sundås Midtvedt

**Affiliations:** † Department of Physics, Chalmers University of Technology, SE-41296 Gothenburg, Sweden; ‡ Department of Physics, University of Gothenburg, SE-41296 Gothenburg, Sweden

**Keywords:** holography, iSCAT, nanoparticles, aggregates, size, morphology

## Abstract

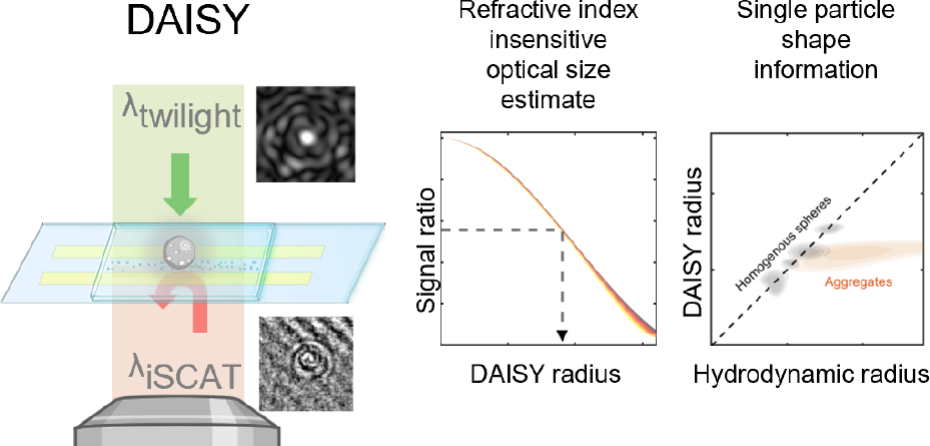

Traditional single-nanoparticle sizing using optical microscopy techniques assesses size via the diffusion constant, which requires suspended particles to be in a medium of known viscosity. However, these assumptions are typically not fulfilled in complex natural sample environments. Here, we introduce dual-angle interferometric scattering microscopy (DAISY), enabling optical quantification of both size and polarizability of individual nanoparticles (radius <170 nm) without requiring *a priori* information regarding the surrounding media or super-resolution imaging. DAISY achieves this by combining the information contained in concurrently measured forward and backward scattering images through twilight off-axis holography and interferometric scattering (iSCAT). Going beyond particle size and polarizability, single-particle morphology can be deduced from the fact that the hydrodynamic radius relates to the outer particle radius, while the scattering-based size estimate depends on the internal mass distribution of the particles. We demonstrate this by differentiating biomolecular fractal aggregates from spherical particles in fetal bovine serum at the single-particle level.

Single-nanoparticle characterization in terms of size, shape, and composition in complex biological environments is a critical challenge within several research areas, including drug delivery,^1^ diagnostics,^2^ and nanosafety.^3^ Optical microscopy is in many cases the tool of choice for studying individual biological nanoparticles due to its high throughput and biological compatibility.^4^ However, although nanoparticles as small as individual proteins can be detected using label-free optical scattering microscopy,^5−8^ multiparametric characterization of individual nanoparticles in terms of properties such as size, refractive index, and morphology remains a challenge.

Since nanoparticles are smaller than the spatial resolution of optical scattering microscopy, it is difficult to estimate their size from direct observation in a microscopy image. Instead, size is typically estimated indirectly by tracking their position over time, estimating their diffusivity from their trajectories, and finally using the Stokes–Einstein relation to relate single-particle diffusivity to the particle size.^9^ However, this requires that the nanoparticles are freely diffusing in a medium with a known viscosity. This imposes critical limitations when the analysis is carried out in the natural environment of the particles (*in situ*) since the viscoelastic properties of biological environments are typically complex and may exhibit spatial variations.^10^

Quantitative *in situ* particle characterization using optical microscopy must instead directly relate the optical scattering of individual particles to their physical properties. The scattering amplitude depends on the particle polarizability, defined as
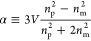
1where *n*_m_ and *n*_p_ are the media and particle refractive indices, respectively, and *V* is the particle volume. Since eq 1 depends on both particle volume and Δ*n*, the scattering amplitude alone is insufficient to characterize both quantities at once.

In addition to the scattering amplitude, the angular distribution of light scattering also contains information about particle size and morphology.^11−13^ This forms the basis of particle characterization using multiangle light scattering (MALS)^14^ and scattering-based flow cytometry.^15^ In the context of microscopy, images of scattering patterns have been employed for simultaneous estimation of size and refractive index of particles with diameters down to about half the wavelength of light.^16,17^ This lower size limit originates from the difficulty of accurately relating a measured scattering image to the particle size for particles near the diffraction limit.

In this work, we introduce dual-angle interferometric scattering microscopy (DAISY), which offers simultaneous quantification of both size and polarizability (and hence also Δ*n*) of individual particles beyond the limits set by diffraction (around half the wavelength of light) without requiring precise information about the surrounding medium. DAISY exploits the fact that although the optical scattering amplitude is related to particle polarizability, the angular distribution of the scattered light is primarily related to the particle size. Thus, by simultaneously measuring the optical signal at two distinct scattering angles, in this work in the forward and backward directions, the particle size can be directly estimated (Figure 1). The forward scattering image is measured using twilight off-axis holography (Figure 1A), which quantifies the complex-valued optical field,^18^ while the backscattering image is measured using interferometric scattering (iSCAT) microscopy (Figure 1B), probing the interference between the backscattered particle signal and a coherent background signal^4^ (Section 1.3, Supporting Information). By measuring twilight holography and iSCAT using two different wavelengths (λ_twilight_ = 532 nm and λ_iSCAT_ = 660 nm), both techniques can be used simultaneously. During a DAISY measurement, the particle positions are detected and temporally linked to form particle traces, from which the scattered light in the forward (twilight holography) and backward (iSCAT) directions is quantified together with the diffusion constant for each detected particle (Figure 1C–E). The twilight holography and iSCAT images are processed using standard algorithms for off-axis holography^19^ and a U-Net trained to generate focused particle images^20^ where the signal is proportional to the scattering amplitude in the backward direction (Section 1.6, Supporting Information), respectively.

**Figure 1:**
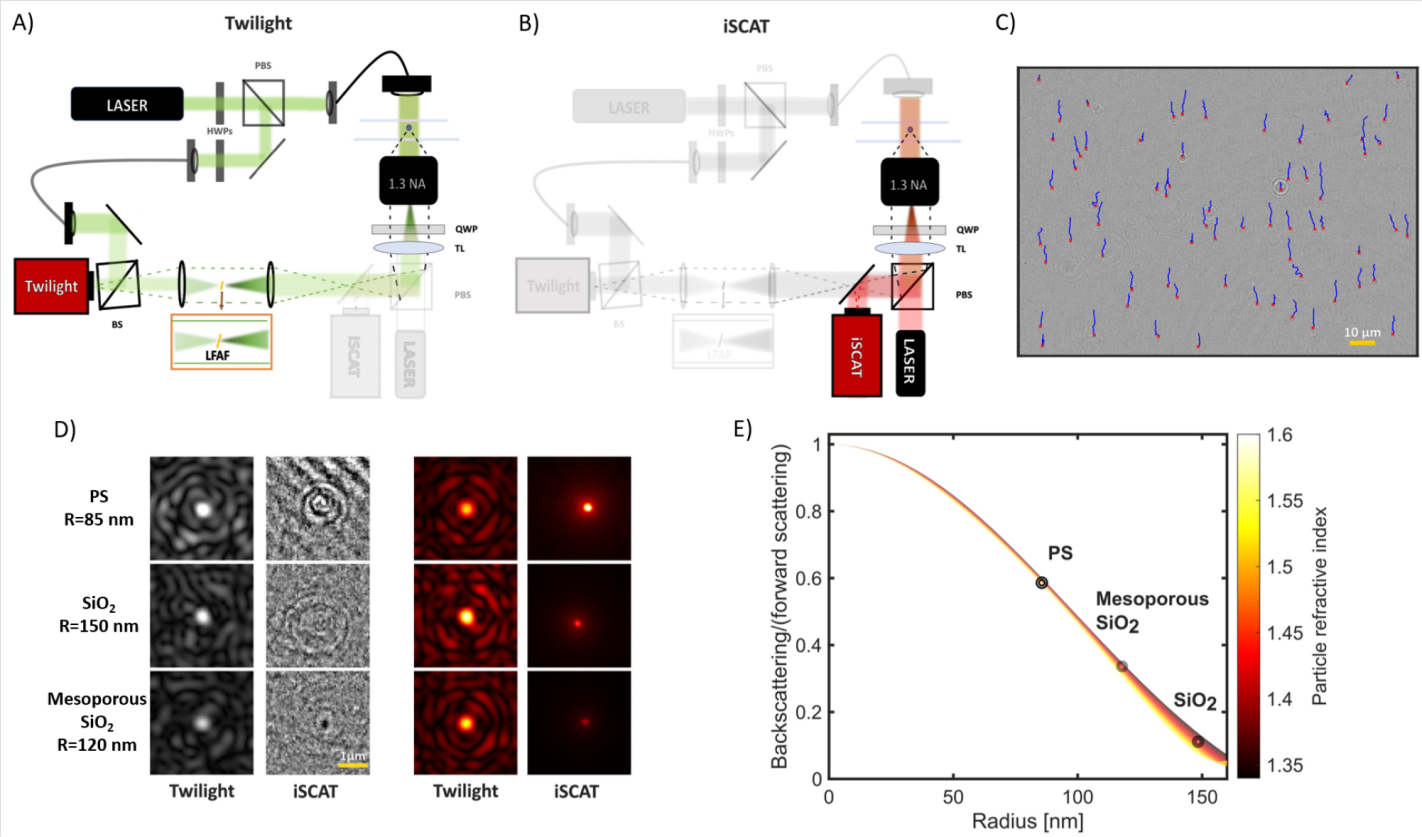
DAISY working principle. (A, B) Optical setup to enable simultaneous twilight off-axis holography (highlighted in A) and interferometric scattering (iSCAT) (highlighted in B) measurements. Using two different wavelengths for twilight holography and iSCAT (λ_twilight_ = 532 nm and λ_iSCAT_ = 660 nm), we separated the two signals by a dichromatic mirror and directed the light to two cameras. The low frequency attenuation filter (LFAF) reduces the amplitude of the unscattered light of the sample beam while having a negligible effect on the particle signal (Section 1.8, Supporting Information).^18,21,22^ The reduction of the unscattered light is highlighted in the zoomed-in inset. The LFAF is slightly tilted to direct the reflected light away from the optical axis. Abbreviations: BS, beam splitter; OBJ, objective; TL, tube lens; QWPs, quarter-wave plates; HWPs, half-wave plates. (C) During a DAISY measurement, the particles are tracked, here exemplified using 105 nm radius polystyrene spheres using the twilight holography data, where for each trace the corresponding local iSCAT and twilight holography images are saved at each observation to be used during the subsequent single-particle characterization. The red crosses are the particle detections, and the blue lines are the ongoing particle traces which are used to estimate the hydrodynamic radii. (D) After detection, the images of particle scattering patterns for each particle trace are postprocessed before the signals are quantified. The two left columns are the scattering patterns after background subtraction, and the two right columns are the same particles but further postprocessed, where the twilight images are the average particle signal along a trace and the iSCAT image was processed using a U-Net. See Section 1.6, Supporting Information, for more information about the postprocessing. Abbreviations; PS, polystyrene; SiO_2_, silica. (E) Ratio between the amplitudes of backscattered and forward-scattered optical fields as a function of particle radius for spherical particles in water. The gradient color scale encodes the dependence of the scattering ratio on the particle refractive index from 1.35 to 1.60. The three circles indicate the size and refractive index of the particles in (D).

On the one hand, the optical field in the forward direction is proportional to the polarizability as (Section 2.3, Supporting Information)^23^
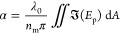
2where λ_0_ is the illumination wavelength in vacuum and  is the imaginary part of the scattered field in the forward direction. On the other hand, the optical signal in the backward direction is proportional to the product between polarizability and the optical form factor *f*, which encodes the interference from different scattering subunits within the particle and describes the difference in signal-size scaling between twilight holography and iSCAT.^11^ Thus, the optical form factor can be experimentally estimated via the ratio between the scattering signals in the forward and backward directions. Within the Rayleigh–Debye–Gans (RDG) approximation, valid for |*n*_p_/*n*_m_ – 1| ≪ 1 and |*n*_p_/*n*_m_ – 1|*kR* ≪ 1, where *k* = 2π*n*_m_/λ_0_ and *R* is the particle radius, the optical form factor depends only on particle size and the refractive index of the surrounding medium and is given by^24^
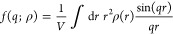
3where *q* = (4π/λ_0_)*n*_m_sin(θ/2), θ is the angular difference between the incoming and scattered light, and ρ(*r*) is the spatial distribution of scattering elements within the particle. Noticeably, within the RDG approximation the optical form factor is independent of the particle refractive index and only weakly depends on the refractive index of the surrounding media (Section 2.4, Supporting Information), which in MALS is used to relate the optical scattering to particle size.^14^

The size range for which RDG theory accurately describes the optical scattering depends on the refractive index difference between the particle and the surrounding media.^11^ In the case of spherical particles, Mie theory can be employed beyond the limitations of RDG, at the cost of introducing a slight dependence of the optical form factor on particle polarizability. This is illustrated in Figure 1E by plotting the ratio between the amplitudes of backscattered and forward-scattered optical fields as a function of the particle radius for spherical particles in water. However, the range of particle sizes and refractive indices corresponding to the same scattering ratio all have different particle polarizability values (Section 1.9.2, Supporting Information). Thus, by using the available single-particle polarizability information in twilight holography, the scattering ratio can be uniquely linked to particle size for particles smaller than 170 nm radius.

To have a unique relation between the measured optical signals and particle size, we define the DAISY radius (denoted by *r*_DAISY_) as the smallest radius of a homogeneous sphere suspended in water, displaying the same backward–forward scattering ratio and polarizability. We here introduce the generalized form factor *f̃* as the theoretical scattering ratio obtained using Mie calculations, normalized such that *f̃*(*R* = 0) = 1 to make it similar to the optical form factor. The scattering ratio measured in DAISY can be related to the generalized form factor as
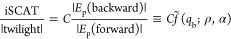
4where *C* is a calibration constant obtained by comparing reference measurements of known particles and *q*_b_ is the effective wavenumber of the iSCAT measurement (Section 1.9, Supporting Information). The generalized form factor is, in turn, related to particle size, where the use of Mie theory instead of RDG theory eliminates the |*n*_p_/*n*_m_ – 1| ≪ 1 requirement for spherical particles.

To validate this approach, spherical particles of different sizes and refractive indices were measured under flow in a microfluidic channel when suspended in water (Section 1.4, Supporting Information). Specifically, two polystyrene samples (*R* = 85 ± 13 nm and *R* = 105 ± 23 nm), one silica sample (*R* = 150 ± 28 nm), and one mesoporous silica sample (*R* = 120 ± 29 nm) were measured (Section 1.1, Supporting Information). Since these particles were measured while diffusing freely in water, the DAISY radius can be compared with the simultaneously obtained hydrodynamic radius (*r*_H_) for each measured particle.

Indeed, the estimated DAISY radius and hydrodynamic radius exhibit a one-to-one correspondence, with a deviation in ensemble median size of less than 5% for all particles (Figure 2A). Moreover, the distribution widths of the DAISY radius are consistently similar to or smaller than the simultaneously obtained hydrodynamic radius from the particle tracking as well as from complementary dark-field nanoparticle tracking analysis (NTA) measurements (Figure S7, Supporting Information). This suggests that the DAISY radius estimation is more precise than the hydrodynamic radius for a fixed track length (Section 1.9, Supporting Information). Consequently, DAISY effectively estimates particle sizes below the diffraction limit in microscopy images without relying on super-resolution imaging or detailed information about the experimental point spread function. At the same time, note that relating the DAISY radius to the outer particle radius requires a known mass distribution within the particle, an assumption that is not needed during diffusivity-based sizing.

**Figure 2:**
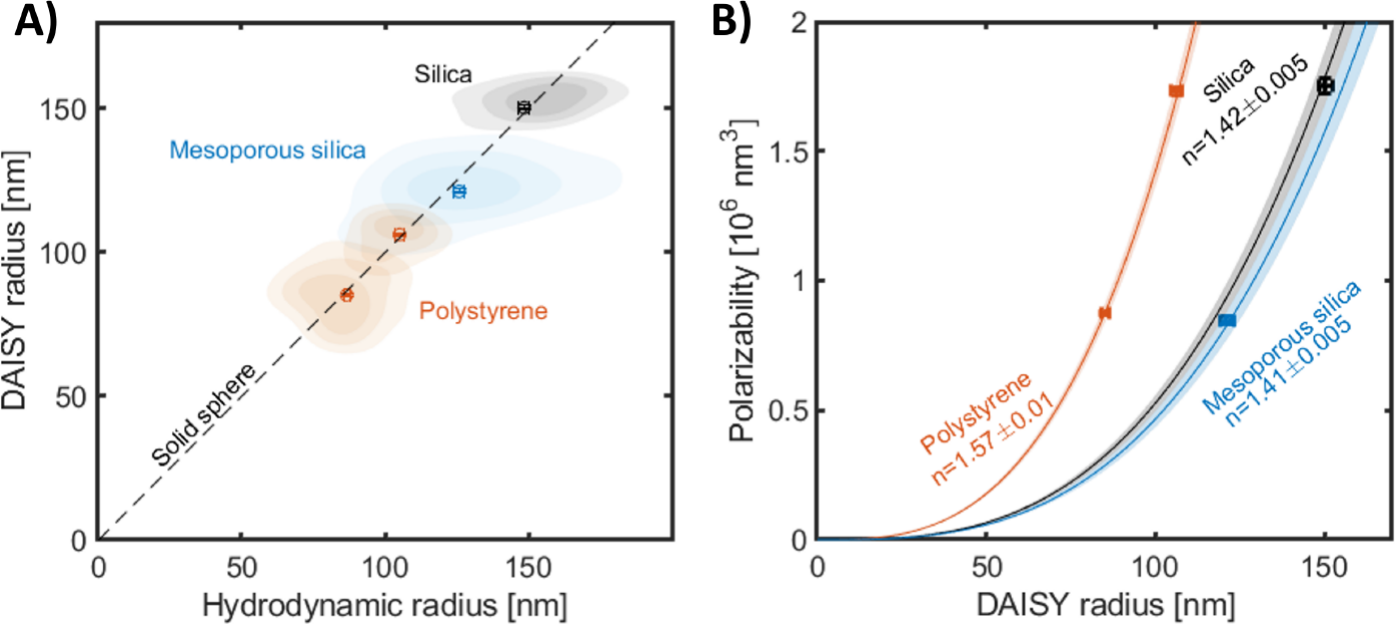
Evaluation of the DAISY radius using the simultaneously obtained hydrodynamic radius as reference. (A) Contour plot of the DAISY radius with the simultaneously obtained hydrodynamic radius for two polystyrene samples, one silica sample, and one mesoporous silica sample, suspended in water. All measured particles follow a one-to-one relationship with the hydrodynamic radius, where the median difference between the two size estimates is less than 5% for all particle populations. The shaded areas correspond to a contour plot of the DAISY radius and the hydrodynamic radius, where the median value is the point in the plots. The contour lines correspond to 50%, 67%, and 83% of the maximum value for the distribution of the measured particles. (B) Refractive index estimates using the polarizability information from twilight holography and the DAISY radius in (A). The ensemble value of the estimated refractive indices is 1.57 ± 0.01 for polystyrene, 1.42 ± 0.005 for silica, and 1.41 ± 0.005 for mesoporous silica, where the solid line is the estimated refractive index value and the shaded region corresponds to the uncertainty in the refractive index estimate.

By using the simultaneously quantified DAISY radius and polarizability, the single-particle refractive index can be estimated, from which the ensemble averaged refractive indices for the measured polystyrene, silica, and mesoporous particles were determined to be 1.57 ± 0.01, 1.42 ± 0.005, and 1.41 ± 0.005, respectively, as depicted in Figure 2B. Notably, all of these values are within a 0.02 refractive index difference from prior estimates.^5,25−28^ This confirms that DAISY enables accurate image-based nanoparticle characterization in terms of size and polarizability without reliance on the Stokes–Einstein relation.

To verify that the DAISY radius is indeed insensitive to the precise information regarding the surrounding media, we measured one particle sample (polystyrene spheres, modal radius 105 nm) in aqueous environments with varying amounts of water and iodixanol, thereby varying the refractive index of the environment (Figure 3). Even though DAISY radius and polarizability are estimated as if the particles are in water, the median DAISY radius for all individually measured particles in each medium remains close to the nominal value of 105 nm and varies by less than 2 nm when the surrounding refractive index is changed from 1.335 to 1.37 (corresponding to a variation in iodixanol concentration between 0% and 24%^29^). This low spread in size estimation is consistent with the observation made in connection with eq 3 regarding that the error in the estimated DAISY radius is bounded by the error in the *q* number used when relating the estimated generalized form factor to the DAISY radius (Section 2.4, Supporting Information). Given the width of the DAISY radius distribution, the spread in median DAISY radius estimates most likely originates from the statistical uncertainty in estimating the median radius rather than any systematic media refractive index dependence. Moreover, the particle polarizability estimation decreases with increasing media refractive index (Figure 3B), as expected from eq 2, since the polarizability is dependent on the refractive index difference between the particle and media. These results indicate that the particle size estimation offered by DAISY remains accurate as long as the relative error in the *q* number is small (≪1).

**Figure 3:**
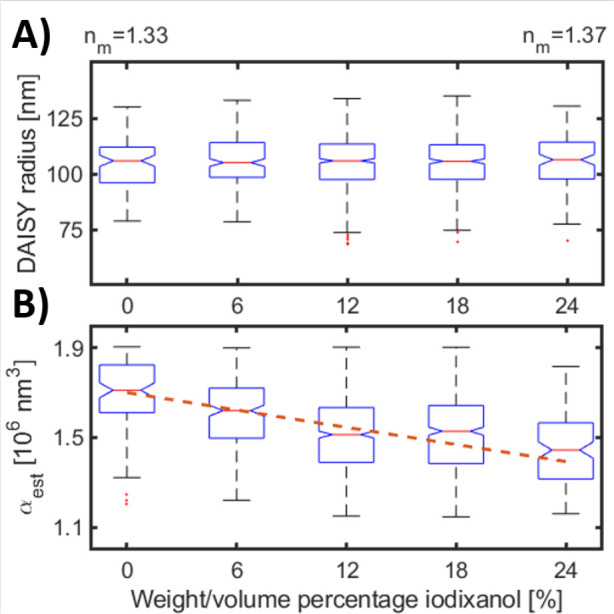
Evaluation of DAISY radius and polarizability determination in different media. Box plots show (A) the DAISY radius and (B) polarizability as a function of the water–iodixanol concentration for 105 nm radius polystyrene spheres. The DAISY radius remains the same for all different media, whereas the polarizability decreases as the refractive index difference to the surrounding media decreases. The DAISY radius and the effective polarizability are here estimated assuming that the surrounding refractive index has the same refractive index as water. The dashed line is the theoretical polarizability for a 105 nm radius polystyrene sphere as a function of the surrounding refractive index.

Note that the DAISY radius is complementary to the hydrodynamic radius, and these two size estimates coincide in the case of homogeneous spheres. To a first approximation, the hydrodynamic radius reflects the physical boundary of the particle, whereas from eq 3 the DAISY radius also reflects the interior mass distribution of the particle. Thus, the relation between the DAISY and hydrodynamic radius can provide information about the spatial distribution of mass within the particle on the single-particle level.

To evaluate the potential of DAISY for estimating particle morphology, we formed aggregates containing 35 nm radius polystyrene spheres via salt-induced aggregation. The detected aggregates deviate from the one-to-one relation between the DAISY radius and the hydrodynamic radius previously observed for homogeneous spheres (Figure 4A), indicating that the morphology of the aggregates is different from that of homogeneous spheres. To develop this observation into a quantitative analysis, we first note that particle aggregates are generally well described as fractal aggregates.^24,30^ Treating the aggregates as spherical units, their mass density is a decaying function of the radial distance from the aggregate center, *n*(*r*) ≈ *r*^*D*_f_/3–1^, where *D*_f_ is the fractal dimension and is expected to fall within the range 1.5–2.3 for particle aggregates.^16,31,32^ An explicit relation between the DAISY radius and overall aggregate radius, here approximated by the hydrodynamic radius, can be derived from theoretical models of fractal aggregates for which the fractal dimension is the only free parameter (Section 2.5, Supporting Information).^24,30^ Since the size range of a unique relation between the optical scattering ratio and particle size depends on particle morphology (Section 2.6, Supporting Information), we found that the (*r*_DAISY_, *r*_H_)-space can be subdivided into two disjoint regions for the sizes in Figure 4A. One of the regions encompasses spherical, nonfractal monomeric units (including homogeneous spheres), whereas the other region encompasses fractal aggregates having fractal dimensions *D*_f_ < 2.7. At this threshold value of the fractal dimension, the theoretical scattering ratio curves for aggregates and homogeneous spheres tangent each other, hindering a reliable separation between fractal and nonfractral structures with fractal dimensions exceeding this value (Figure S9, Supporting Information). Furthermore, each point within the fractal aggregate region is related to a specific value of the fractal dimension, indicating that the fractal dimension of individual aggregates can be quantified based on their position in (*r*_DAISY_, *r*_H_)-space. We found that the salt-induced aggregates generally fall within the region in (*r*_DAISY_, *r*_H_)-space encompassing fractal aggregates, validating the analysis approach outlined above (Figure 4A). The polystyrene aggregates have a population-wide median fractal dimension of *D*_f_ = 2.0, consistent with the expectation for diffusion-limited cluster aggregation (inset to Figure 4A).^31,32^

**Figure 4:**
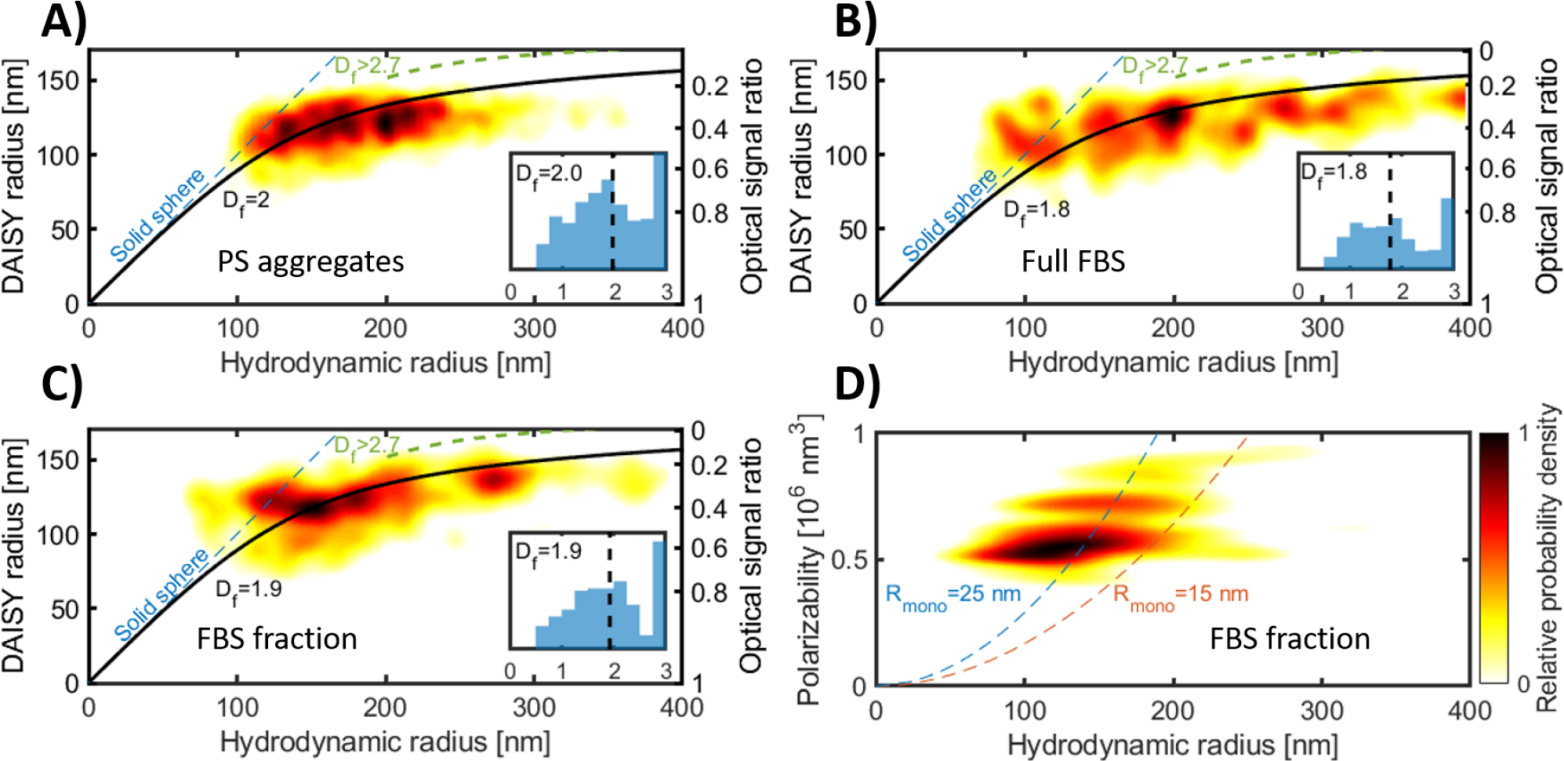
DAISY radius and hydrodynamic radius to distinguish particle aggregates from solid spheres. (A) DAISY radius as a function of hydrodynamic radius for salt-induced aggregation of 35 nm radius polystyrene particles. From the comparison with the theoretical lines, the DAISY-hydrodynamic radius relation agrees with that of fractal aggregates with a fractal dimension of around 2.0. (B, C) The DAISY radius and hydrodynamic radius for freeze–thawed induced aggregates of fetal bovine serum (FBS), both (B) in full serum and (C) after size-exclusion chromatography. In (A)–(C), the curved lines correspond to the theoretical relation for fractal aggregates and the straight dashed line is the expected scaling for a solid sphere. The green dashed lines correspond to a fractal dimension of 2.7, which separates true aggregate detections from that of spheres with a hydrodynamic radius larger than 200 nm. The insets are single-particle fractal dimension histograms, where the dashed line corresponds to the median fractal dimension value. The number of particle detections in FBS corresponds to a concentration of around 10^8^/mL. (D) Polarizability as a function of hydrodynamic radius for the data in (C), where the dashed lines are the expected scaling for aggregates with a fractal dimension of 1.9 and a monomer refractive index of 1.5. The color scale in all plots describes the relative probability density of the single-particle observations.

To investigate whether the same analysis approach can provide information about the morphology of the constituents in more complex solutions, we performed measurements of freeze–thawed fetal bovine serum (FBS), both nontreated and depleted of proteins (Section 1.2, Supporting Information). In addition to individual dissolved biomolecules, FBS contains biological particles such as extracellular vesicles (EVs), lipoprotein particles, and protein aggregates.^33^ Using DAISY, we detected particles with a hydrodynamic radius between 100 and 400 nm in both ordinary FBS and FBS separated from free proteins using size-exclusion chromatography (Figure 4B,C) at a concentration of about 10^8^/mL. Notably, DAISY’s detection count was 10^3^ times lower than dark-field measurements (Figure S1, Supporting Information), suggesting that it primarily identifies larger particles or aggregates above its detection limit. The particle detections with a hydrodynamic radius of around 150–200 nm have a fractal dimension close to *D*_f_ = 2.0, whereas the larger particles have a fractal dimension in the vicinity of *D*_f_ = 1.7. In addition to this, a small fraction of detections with a hydrodynamic radius around 100–150 nm deviate from the fractal aggregate scaling, in particular for the FBS after size-exclusion chromatography, and instead coincide with the expected scaling for homogeneous spheres (Figure 4C). These detections have a polarizability of around 0.55 × 10^6^ nm^3^, which together with a hydrodynamic radius of 125 nm corresponds to a refractive index of about 1.38 (Figure 4D). This value is similar to the expected values for EVs,^27,34,35^ which, if filled with biological material, are expected to have an optical form factor similar to that of homogeneous spheres. However, identification of the EV surface markers is required for conclusive identification of the presence of EVs. Nevertheless, the rich single-particle shape information using DAISY indicates that it enables analysis of subpopulations and heterogeneity within the sample, which extends the possibilities compared to previous works where particle shape is estimated on the ensemble level from the signal-size scaling.^5,16,36^

To gain additional insights into the nature of the fractal aggregate population of FBS, we investigated the relation between polarizability and the hydrodynamic radius for FBS after size-exclusion chromatography. The polarizability of a fractal aggregate is directly proportional to the number of monomers *N* in the aggregate as α = α_0_*N*, where α_0_ is the polarizability of the monomers. Since the hydrodynamic radius also scales with the number of monomers and the fractal dimension is known from the relation between DAISY radius and hydrodynamic radius, we can estimate the monomer polarizability. Assuming that the monomer has a refractive index of 1.5, which is similar to lipid bilayers, proteins, and lipoprotein particles,^35,37,38^ we find that the monomer has a hydrodynamic radius of around 20–30 nm (Figure 4D). This value is considerably larger than individual proteins (having a typical radius of less than 10 nm).^39^ The estimated properties of the aggregate are thus consistent with a larger monomer, with lipoproteins being a likely candidate.^35^ However, viral or EV monomers cannot be excluded based on this data alone.^35,40^ It should be noted that these results do not exclude the presence of protein aggregates with smaller monomer units in FBS; they demonstrate that the aggregates that were detected in our setup consist of monomers of this size.

In conclusion, we have introduced a versatile method for multiparametric particle characterization, namely, dual-angle interferometric microscopy (abbreviated DAISY), based on the simultaneous quantification of forward-scattered and backscattered light from individual nanoparticles combined with single-particle tracking. We have demonstrated the capacity of DAISY to simultaneously quantify the radius and polarizability of particles directly from optical scattering patterns without being limited by the diffraction limit for particles smaller than 170 nm. However, for particles larger than this limit the forward-scattered light is sufficient to perform accurate sizing,^16^ indicating potential optical particle sizing from the sub-100 nm regime to several micrometers. Moreover, the DAISY radius shows a negligible dependence on the refractive index of the surrounding media and is complementary to the hydrodynamic radius, allowing the combination of the DAISY and hydrodynamic radii to provide particle morphology estimates. Thus, DAISY opens up for multiparametric analysis for both suspended particles and of nanoparticles in biological environments, extending what is possible using holography and iSCAT as separate techniques.^41,42^ Note also that current precision in size estimation is limited by statistical uncertainty from having an average track length of around 50–100 frames, where a higher frame rate will significantly improve the precision of the particle sizing. Moreover, kHz imaging would also enable analysis of nonrotational asymmetric particles based on the fluctuations in the measured scattering signal,^43^ which could be used to further the shape analysis of DAISY. Given DAISY’s versatility and the presented characterization opportunities, we anticipate that this type of optical-microscopy-based multiparametric characterization will find widespread application in many areas where nanoparticles play an important role, ranging from industrial processes to drug discovery and medical diagnostics.

## Supporting Information Available

Materials and methods, data analysis, and theoretical descriptions of the DAISY radius and the optical scattering ratio for fractal aggregates. (PDF)
