# Supplementary material for: Dual-Angle Interferometric Scattering Microscopy for Optical Multiparametric Particle Characterization
Source: Nano Lett. 2024 Jan 31;24(6):1874–81. doi: 10.1021/acs.nanolett.3c03539 (PMC10870763; doi:10.1021/acs.nanolett.3c03539)
Supplement: Supplementary file 1 — nl3c03539_si_001.pdf [file nl3c03539_si_001.pdf]

Supporting Information
Dual-angle interferometric scattering microscopy for optical multiparametric particle characterization

Erik Olsén[1*], Berenice García Rodríguez[2], Fredrik Skärberg[2], Petteri Parkkila[1], Giovanni Volpe[2], Fredrik Höök[1], and Daniel Sundås Midtvedt[2*]

[1]Department of Physics, Chalmers University of Technology, SE-41296 Gothenburg, Sweden
[2]Department of Physics, University of Gothenburg, SE-41296 Gothenburg, Sweden

January 22, 2024

* Email: erik.olsen@msl.ubc.ca, daniel.midtvedt@physics.gu.se

Contents

1 Materials and methods 2
1.1 Particles . . . 2
1.2 Fetal bovine serum . . . 2
1.3 The microscope setup . . . 3
1.4 Experimental conditions . . . 3
1.4.1 Salt-induced aggregation . . . 4
1.4.2 Iodixanol series . . . 4
1.5 Overview of the data analysis pipeline . . . 5
1.6 Image processing . . . 5
1.6.1 U-Net for iSCAT processing . . . 7
1.7 Particle tracking and diffusivity estimation . . . 8
1.7.1 Filtration of particle tracks . . . 9
1.8 Optical signal quantification . . . 9
1.8.1 Z-dependence of the optical signals . . . 10
1.9 Quantification of the DAISY radius . . . 10
1.9.1 Comparison in precision of the DAISY radius . . . 11
1.9.2 The single-valued relation to DAISY radius . . . 13

2 Relating the optical signal to particle properties 13
2.1 Generalization of the optical form factor using Mie . . . 13
2.2 iSCAT particle signal . . . 14
2.3 Twilight holography particle signal . . . 15
2.4 Media refractive index dependence in particle scattering from Mie theory . . . 15
2.5 Optical form factor of fractal aggregates . . . 16
2.6 DAISY for larger particles . . . 17
2.7 Practical lower size limit for DAISY . . . 17

1 Materials and methods

1.1 Particles

The used particles are 105 nm (modal) radius polystyrene beads (Sigma-Aldrich), 85 nm (modal) radius polystyrene beads (Sigma-Aldrich), 120 nm (modal) radius mesoporous silica (Sigma-Aldrich), 150 nm (modal) radius Silica particles (KISKER BIOTECH GmbH & Co.KG), 35 nm (modal) radius amidine latex beads (Thermo Fisher Scientific), and 228 nm (modal) radius (NIST-certified standard deviation ±6.8 nm) polystyrene (Polysciences), where the sizes were verified using nanoparticle tracking analysis in scattering mode (NanoSight LM10 module, Malvern Instruments Ltd., United Kingdom; 488 nm laser).

1.2 Fetal bovine serum

Fetal bovine serum (Gibco) was aliquoted and stored at -20 °C until use. Serum was thawed at +4 °C and centrifuged at 300 g for 10 minutes (+4 °C). 500 µL of the supernatant was collected and applied to a miniPURE-EV size exclusion chromatography column (HansaBioMed Life Sciences) equilibrated with 2 column volumes (2 × 4 mL) of 0.2 µm-filtered phosphate-buffered saline (PBS, Sigma-Aldrich). Eighteen fractions of approximately 200 µL volume were collected. Selected fractions were analyzed for particle concentration using nanoparticle tracking analysis in scattering mode, and all fractions were analyzed for protein concentration using Pierce bicinchoninic acid (BCA) protein assay kit (Thermo Fisher Scientific). Particle and protein concentrations of the different fractions are shown in Figure S1. As fraction 8 had the highest particle concentration, it is the fraction shown in Figure 4c in the Main text. Since the protein concentration is close to zero for that fraction, the viscosity is assumed to be the same as the buffer (PBS). For the non-diluted serum, the viscosity at 20°C was measured to 1.1 mPas (Lovis 2000 M, Anton Paar).

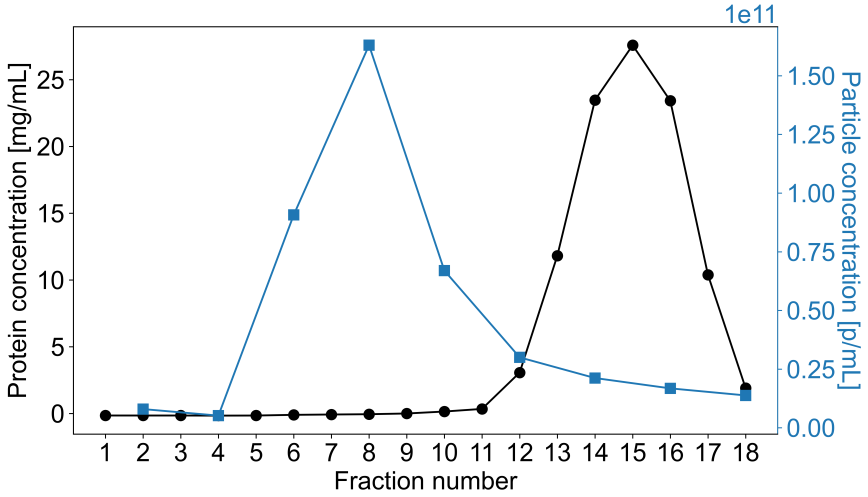

Figure S1: **Protein and particle concentration of the fetal bovine serum after size exclusion chromatography**. The particle concentration is estimated using darkfield nanoparticle tracking analysis (blue squares), whereas the protein concentration is estimated using a protein assay kit (black circles). The particle and the protein concentration peaks are well separated, indicating that the size exclusion chromatography separates the particle and protein fractions.

1.3 The microscope setup

The microscope is custom built using a 40x oil-immersion objective with a numerical aperture (NA) of 1.3 (Olympus). The iSCAT illumination consists of an OBIS 660 nm LX 75 mW laser operated at 20% power, and the twilight off-axis holography illumination consists of a continuous 100 mW $\lambda = 532$ nm diode-pumped solid-state (DPSS) laser (Roithner Lasertechnik GmbH), where the lasers are separated using a short-pass dichroic mirror with 567 nm cut-off wavelength (Thorlabs). The image recording was done using a customized LabVIEW programme which synchronized the recording between the two cameras.

The twilight off-axis holography microscope is similar to Ref. [1]. In off-axis holography, the laser beam is split into two beams, where the intensities of the two beams are controlled by a $\lambda/4$ plate and a polarization-dependent beam splitter. After the polarizing beam splitter, these beams are focused into two optical fibers, which guide the light to the sample and reference beam path. One of the optical fiber ends is positioned just above the sample. The low frequency attenuation filter (LFAF) [2, 3] is a gold disc with a thickness and diameter of 105 nm and $\sim$500 µm, respectively, which is located at the intermediate focal plane of a 4f system for the light beam that has interacted with the sample (Section 1.7). The second laser beam is expanded to a few centimetres in diameter before both beams are recombined before the camera, ProSilica GX1920 (AlliedVision), at a slight offset angle using a beam splitter, where the offset angle separates the interference terms when the recorded image is Fourier transformed. The effective pixel size of the twilight holography camera is the same as in Ref. [1], which was obtained by measuring the diffusivity of NIST-certified polystyrene particles.

The iSCAT microscope is similar to conventional iSCAT widefield microscopes [4]. The incoming and outgoing light is separated using a polarization beam splitter and a quarter waveplate (Figure 1 in Main text), where the camera is an ORCA-Flash 4.0 V2.0 CMOS camera (Hamamatsu).

The LFAF for the twilight holography was made in the cleanroom at Chalmers University of Technology. A circular cover-slip underwent a dehydration bake consisting of 5 minutes on a hotplate at 190-200 °C. The cover-slip was spin coated with LOR 1A at 3000 rpm for 45 seconds, followed by prebaking on a hotplate (5 minutes at 190-200 °C). It was thereafter spin coated with S1813, 3000 rpm for 45 seconds, and prebaked on a hotplate (5 minutes at 110 °C). The sample was then exposed using a laserwriter and developed in MFCD 26 for 60 s, followed by rinsing in DI water and descum using oxygen plasma. The metal was thereafter deposited using e-beam evaporation; first, 3 nm titanium and then 105 nm gold, followed by lift-off in 1165 remover for approximately 100 minutes and subsequent rinsing with isopropanol and deionized water.

1.4 Experimental conditions

Each measurement is performed using a sample volume of around 10 µL placed in the inlet of a custom-built straight microfluidic chip, consisting of two glass slides spaced using 30 µm double sided tape (Figure S2a), where the flow was either gravitationally controlled or by using tissue paper at the outlet, where the latter was used during the water-iodixanol series. To minimize the risk of particles sticking to the wall of the microfluidic channel, each measurement series starts incubating the channel with a 5 mg/ml bovine serum albumin (BSA) solution (Sigma-Aldrich) for at least five minutes. The frame rate is 41 frames per second, the exposure time is between 3-4 ms for the twilight holography images and 2 ms for the iSCAT images, and each recorded video is around 2000-3000 frames. The room temperature was 22 °C. For all particles included in the shown figures, a minimum track length of 20 frames was used, while the flow

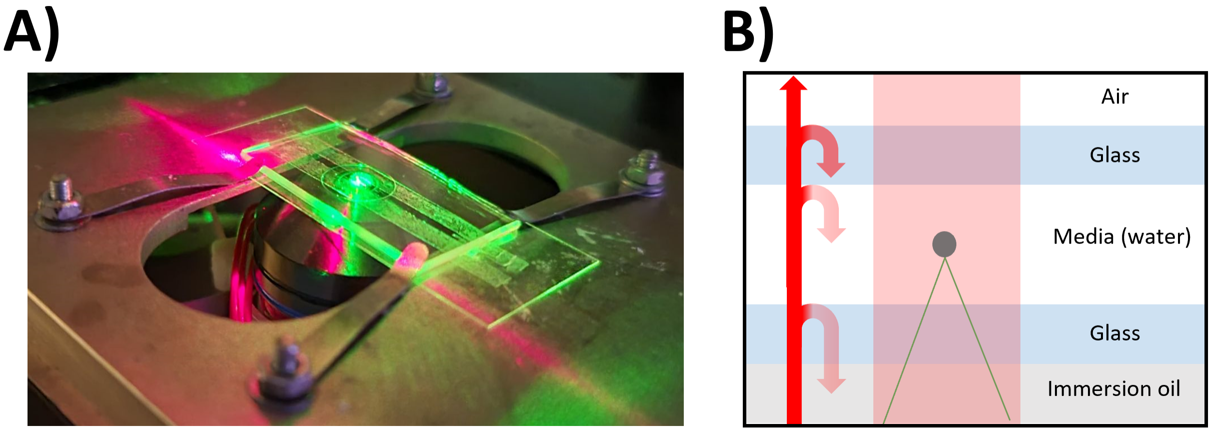

Figure S2: **The microfluidic chip and the surface reflections in iSCAT**. **A** Image of the costume built microfluidic setup, consisting of two glass slides spaced using 30 µm double sided tape. **B** When using microfluidics with iSCAT, there are several back reflections that will interfere with the particle signal. Specifically, where there are two glass-media reflections and a glass-air interface.

speed of the particles during the measurements was adjusted such that the average track length was around 50-100 frames.

The focal plane in the measurements was chosen to be in the middle of the channel for each measurement, where the cameras were aligned to share the same focal plane. This image plane was found by that the particles in the middle of the channel have the highest flow speed in the microfluidic channel.

Due to the use of microfluidics chips, several back reflections interfere with the particle signal in iSCAT (Figure S2b). Specifically, there are reflections from the two glass-media interfaces and the glass-air interface. The reflection from the glass-air interface was handled by having a slight angle between the incoming illumination and the optical axis of the microscope, which separates the glass-air reflection from the channel reflections as the glass slides are much thicker than the depth of the channel. The two media-glass reflections were handled using an iSCAT laser with a coherence length much greater than the height of the channel, and that the height in the used microfluidic chip varied slightly between different regions in the channel. The height variations affect the relative phase between the two reflections, resulting in constructive/destructive interference depending on the chosen imaging position in the channel. The imaging position was chosen such that the two reflections interfered constructively.

1.4.1 Salt-induced aggregation

The stock solution (4% w/v) of 35 nm (modal) radius amidine latex beads was diluted 500 times in MilliQ water. To that, 20 µL of saturated NaCl solution was added and mixed, and after 5 seconds, 1 ml of MilliQ water was added to slow down the aggregation. The mixture was then directly injected into the microfluidic chip, where the time from dilution to measurement was 2-3 minutes.

1.4.2 Iodixanol series

OptiPrep (Sigma-Aldrich), which contains 60% (w/v) iodixanol, was diluted with PBS to obtain media with 0%, 6%, 12%, 18%, and 24% (w/v) iodixanol. The relation between iodixanol concentration and the refractive index was obtained from Ref. [5].

1.5 Overview of the data analysis pipeline

An overview of the data analysis for the measurements is presented in Figure S3, where the details are further presented in Sections 1.6-2.7. Note that all of this analysis is done on the single particle level.

First, the images are pre-processed, which includes background subtraction and isolating the optical field in the twilight holography image (Section 1,6), and the particles are tracked using their signal in twilight holography (Section 1.7). The single particle diffusivity and hydrodynamic radius are estimated from these particle traces, where the position uncertainty is estimated on the ensemble level (Section 1.7).

During the tracking, for each particle observation along the trace, a particle centered 64×64 cropped image of the particle is saved. The size of the cropped image is chosen such that it is large enough to fully contain the scattering pattern of the particle, even if it is a bit out of focus, but also small enough such that it only contains a single particle. The single particle twilight holography images are then numerically refocused using the criteria presented in Section 1.7. A cropped image from the same particle position using the iSCAT image is also added to the particle trace. Thus, the iSCAT and twilight holography information are obtained for all measured particles throughout the particle trace.

After linking the twilight holography and iSCAT single particle observations, the particle signal measured using both twilight holography and iSCAT is quantified (Sections 1.6.1 and 1.8). After the image processing, which generates focused particle images, the particle signal is estimated by Gaussian fitting. The polarizability is estimated from the twilight holography images (Section 2.3).

Thereafter, the DAISY radius is estimated using the combined information from twilight holography and iSCAT by relating the scattering ratio to the generalized form factor, where the details are presented in Section 1.9. In short, by considering both the scattering ratio and the polarizability there is a unique relation between the measured particle information and size when assuming the particle shape.

Once the DAISY radius is estimated, the single particle refractive index is calculated using the DAISY radius and polarizability. This is estimated using the expression for polarizability ($\alpha \equiv 3V\frac{n_\mathrm{p}^2-n_\mathrm{m}^2}{n_\mathrm{p}^2+2n_\mathrm{m}^2}$) where the particle volume is estimated using the DAISY radius under a homogeneous sphere approximation.

After estimating the DAISY radius, hydrodynamic radius, polarizability, and refractive index, the particle estimates can, in some cases, also be used to calculate additional particle properties such as particle shape. The details of this are presented in Sections 2.5-2.6

1.6 Image processing

All data analysis except for the training of the U-Net was performed using MATLAB (Mathworks Inc.). The holograms were analyzed using customized code to extract the amplitude and phase maps using standard methods [6]. In brief, due to the off-axis configuration of the setup, the Fourier transform of the interference pattern contains two off-center peaks, which consist of the complex-valued object signal multiplied by a plane wave ($\exp(\pm i\vec{k} \cdot \vec{R})$), in addition to the central peak which corresponds to the non-interferometric intensities. To isolate the object signal, we numerically shifted one of the off-centre peaks to the center of the Fourier image (multiplying the recorded image by $\exp(\pm i\delta\vec{k} \cdot \vec{R})$) and applied a low-pass filter. The phase background was corrected by fitting the phase of the field to a fourth-order polynomial and then subtracting the fit from the image. The optical signal was after that normalized such that the background was centred around 1. The static background signal was removed by sub-

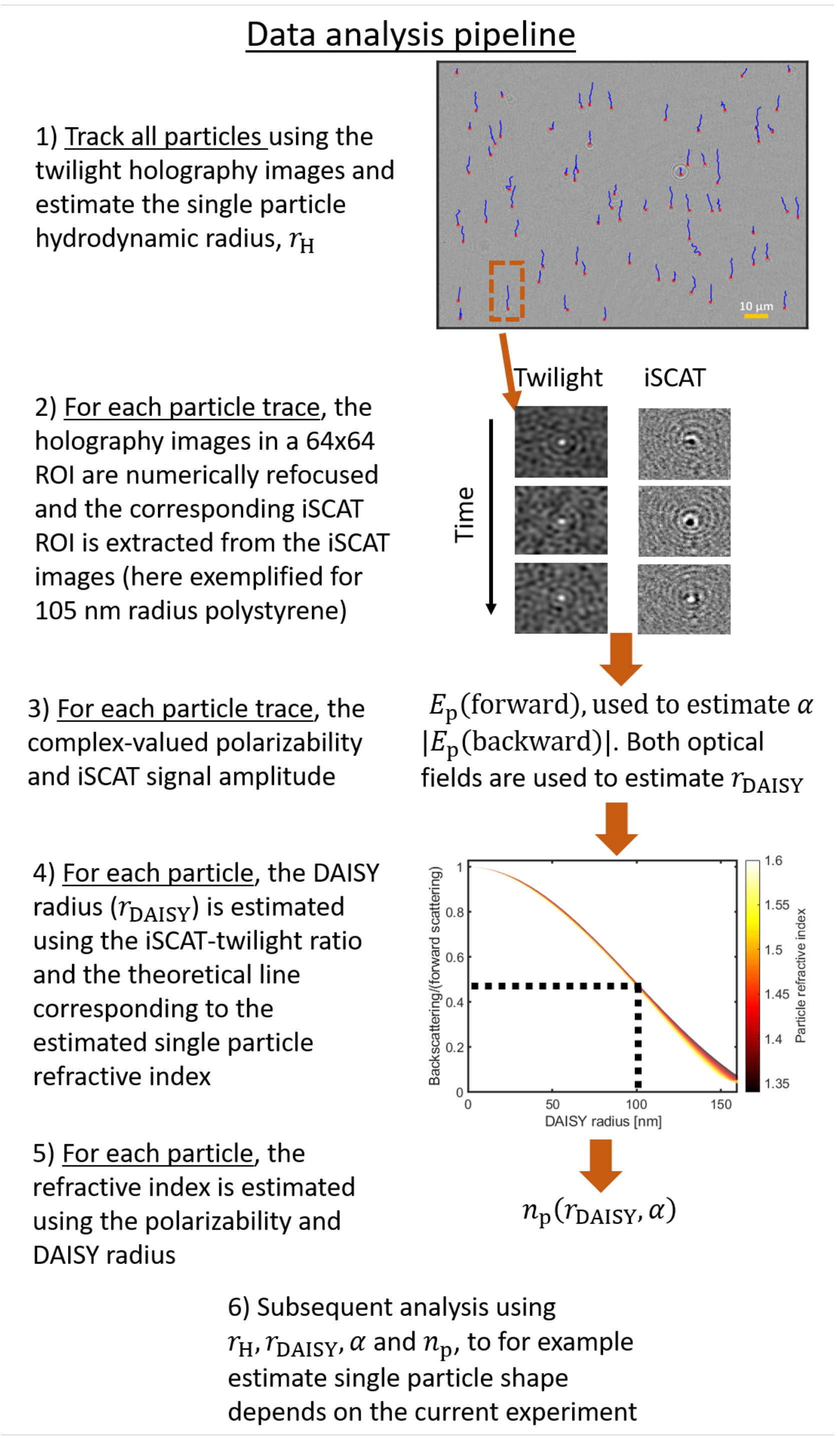

Figure S3: **Overview of the data analysis pipeline**. 1) The particles are tracked using twilight holography. 2) For each particle trace, 64 × 64 cropped images of the particle are added to the particle position information. The twilight holography image is also numerically refocused. A cropped image from the same particle position in the iSCAT data is added to the particle trace. 3) The particle signal measured using twilight holography and iSCAT, and the polarizability is estimated from the twilight holography images. 4) The DAISY radius is estimated using the combined information from twilight holography and iSCAT. 5) The single particle refractive index is estimated using the DAISY radius and polarizability. 6) Subsequent analysis of, for example, particle shape depends on the current experiment. Note that all the analysis is done on the single particle level.

tracting the image using a set of background images, from which the image combination that lowered the subtracted image's standard deviation the most was chosen. The list of potential background images was constructed from a set of fields in proximity but not direct temporal adjacency to the target field (specifically, for target frame "i" we consider the reconstructed fields from frames "i-50" to "i-3" and "i+3" to "i+50")

Each iSCAT image was first transformed and aligned using the Procrustes algorithm to overlay the two imaging modalities. The transformation was identified by manually identifying the position of the same particle in both images for approximately 20 different particles. The iSCAT images were then normalized such that the median value is equal to 1, and the static background signal was subtracted as for the twilight holography, where a set of iSCAT images in proximity but not direct temporal adjacency to the target image was used. After that, for each particle detection in twilight holography, a 64×64 region of the same position in iSCAT was saved into a trace. The median of each 64×64 region was then subtracted, and each image was normalized by its standard deviation before further image processing using the U-Net.

1.6.1 U-Net for iSCAT processing

The network used is based on the U-Net architecture [7], and its details are shown in Figure S4. Aside from the information given in the figure caption, the input is an image of size (64×64), and the network has in its up- and downsampling path $\{16, 32, 64, 128\}$ filters in the base block $\{128, 128\}$ filters and in the last step $\{16, 16\}$ filters. In total, the network contains 897,809 trainable parameters.

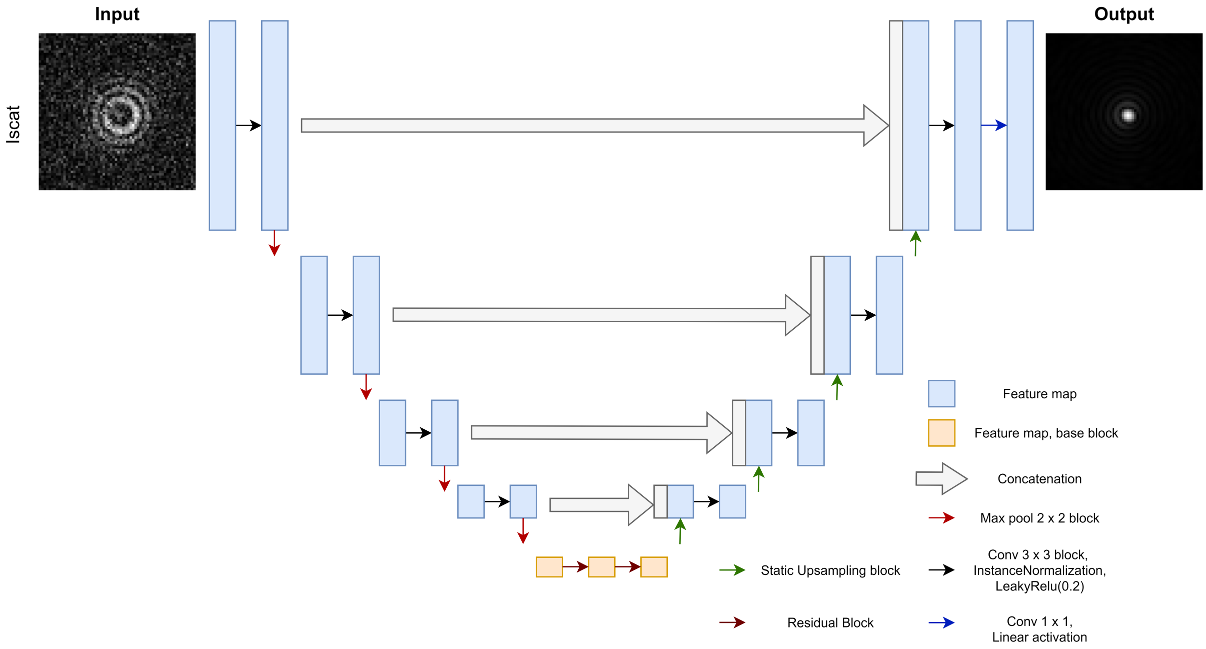

Figure S4: **U-net architecture**. The U-Net architecture comprises of four downsampling and upsampling blocks. The downsampling path has convolutional blocks with a 3x3 kernel and Relu activation followed by Instance Normalization and 2x2 downsampling. In the upsampling path, there are transposed convolutional blocks with a 3x3 kernel and Relu activation followed by Instance Normalization. Within the base block, convolutional blocks are executed, and a linear activation is applied in the final convolutional layer.

The input to the network is an iSCAT image of size (64×64), where the initial image processing is described in Section 1.6.1. To perform such simulations, we utilize DeepTrack

2.0 [8], a software package developed for simulations of optical systems and training of neural networks. The training is based on Mie simulations of the backscattered light where the particles are placed with ±1.5 µm from the center of the image (to correct for imperfections with image overlay), depth position between ±7.5 µm, a radius ranging from 50-200 nm, a particle refractive index between 1.35-1.60, a medium refractive index of 1.33 and a vacuum wavelength of 660 nm. These values capture the range of particle sizes and refractive indices of the particles included in the analysis. The relative phase between the particle and background field was random to mimic the depth dependence of the iSCAT signal. The input image is also augmented such that its noise level is close to that in the experimental data, where the resolution matches the microscopy images.

The network output consists of a particle image in focus, with minimized noise, corresponding to the absolute value of its backscattered optical field when the particle is in focus. This output is then multiplied with the same standard deviation value used to normalize the input data to the U-Net. An example of this input-output pair is shown in the top left and right of Figure S4.

Training a neural network involves feeding data through a network, adjusting the weights to minimize the error via backpropagation, and repeating this process for multiple epochs. Our network receives data via a generator that generates new data samples for each batch, leveraging the above simulation pipeline. For the training of the network, we employ mean absolute error (of the pixels) as our loss function,

$$\mathcal{L}_{\text{mae}} = \frac{1}{N}\sum_{i=1}^{N} |x_i - y_i|, \tag{1}$$

where x_i represents the label and y_i the prediction, which in this case are two images. Furthermore, the Adam optimizer [9] is chosen with a learning rate of $5e - 4$, and $\beta_1 = 0.9$ and $\beta_2 = 0.999$. The training is done with a batch size of 32 until the L_{mae} no longer improves. The training is done via the DeepTrack 2.0 package, with Python 3.9.7 and Tensorflow 2.6.0 [10]. The hardware configuration features an NVIDIA GeForce RTX 3090 GPU, an Intel Core i9 10900KF processor, 64 GB of memory, and runs on Windows.

1.7 Particle tracking and diffusivity estimation

In-plane subpixel localization and linking between subsequent observations are based on the twilight holography data, where the used algorithms are the same as in [1]. In short, a depth stack of complex-valued optical images was projected onto a 2D plane, where in-plane subpixel localization of detected local extreme values of the signal amplitude was performed using the radial center method [11]. The first depth localization along a particle trace is based on minimizing the standard deviation of the Fourier image of single particle detection, which assumes point-like particles [12]. Subsequent depth localization estimates are based on minimizing the standard deviation between the Fourier image of the initial particle observation and the Fourier image of the current particle observation.

The hydrodynamic radius of the tracked particles was estimated using the Stokes-Einstein relation (at temperature 22 °C). The diffusivity is estimated using the twilight holography data and the in-plane particle movement orthogonal to the flow direction. To accurately relate mean squared displacement to diffusivity and hydrodynamic radius, the contribution from the position uncertainty needs to be removed [13]. The contribution from position uncertainty was corrected by estimating the average position uncertainty for particles included in the analysis, where that average value was used to correct the mean squared displacement estimation for individual particle traces.

1.7.1 Filtration of particle tracks

A few different filtration steps were used to minimize the risk of including false traces in the final analysis. First, the minimal track length was 20 frames. Second, the mean step length in the flow direction between subsequent frames needed to be larger than approximately 2 pixels ($\sim$ 230 nm). Third, the mean step length in the flow direction of the channel was at least ten times larger than the absolute value of the mean step length orthogonal to the flow direction. Fourth, the goodness of fit for a 2D Gaussian to the absolute value of the particle signal in both twilight holography and iSCAT needed to be larger than 0.5. Fifth, the particles need to have an average z-position within five microns of the center of the channel (Section 1.8.1).

Criteria two and three come from the fact that the particles are measured during flow. The flow speed is set so that it takes around 2 to 5 seconds for a particle to traverse the field of view, corresponding to an average step length of approximately 4 and 18 pixels. Moreover, the relation between the step length in the flow direction and the orthogonal direction eliminates most potential noise traces. The criteria of goodness of fit further eliminates the risk of including noise traces, and the z-selection minimizes the effect from a weak z-dependent particle signal (Section 1.8.1). The first four criteria exclude around 10-20% of the traced particles from the analysis, whereas the fifth criterion exclude a bit more than half of the traced particles as the included z-range is three times smaller than the depth of the microfluidic channel.

1.8 Optical signal quantification

Using an LFAF reduces the background signal in the holography image without affecting the particle signal [1]. This allows for increased illumination power without saturating the image, increasing the amount of light scattered by each particle compared to off-axis holography and improving the detection limit. However, the presence of the LFAF affects the relation between measured particle signal and particle properties since the particle signal is normalized to the background signal, where the effect needs to be compensated to accurately relate the signal particle properties [1]

Since the LFAF selectively attenuates the unscattered light, the optical signal at the camera becomes

$$E_s = aE_0\left(1 + (E_{\mathrm{p}}/aE_0)\right), \tag{2}$$

where $|a| < 1$ is a complex constant that encodes the effect of the LFAF. In the absence of the LFAF, $a \equiv 1$. From this relation, it is clear that the LFAF perturbs the interferometric signal $(1 + (E_{\mathrm{p}}/aE_0))$ that is measured in off-axis holography. To quantify particle properties such as polarizability from the scattered field E_{p} from an particle, it is therefore necessary to determine the parameter a.

The conversion factor a was obtained by measuring a sample of 105 nm median radius polystyrene beads with and without the LFAF. The particle suspension was diluted in MilliQ until approximately 100 particles were in the field of view. The search for that factor was performed using the function `lsqnonlin` in Matlab, with the criteria that the media optical field should be the same after the compensation. The residuals of the fit were used to estimate the uncertainty. The obtained conversation factor is $(0.0688 \pm 0.0024)\exp\Big(-i(0.895 \pm 0.036)\Big)$ (Figure S5), which corresponds to an improved signal-to-background ratio of around 14 times.

The particle signal is estimated by evaluating the spatial integral of Gaussian fits to the final post-processed images. In twilight holography, the fit is done using the average particle field for all observations along the trace. In iSCAT, the fit is done for each image, where for each trace, the 10 fits with the best goodness of fit are used to estimate the particle signal.

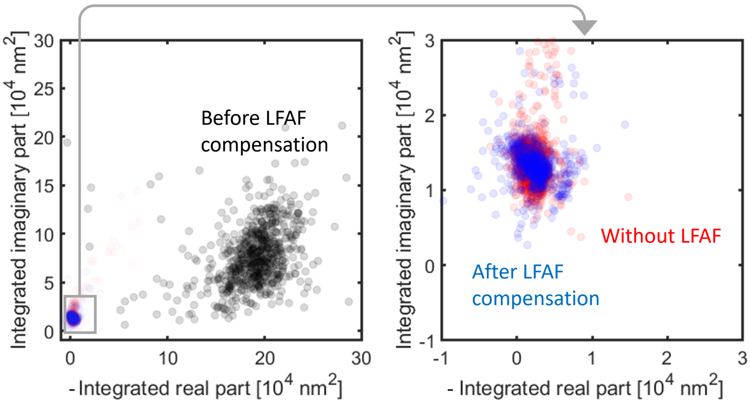

Figure S5: **Compensation of the low frequency attenuation filter (LFAF).** A 105 nm radius polystyrene particle sample is measured with and without the LFAF present. The similarity with and without the LFAF indicates the effect of the quantified optical signal can be compensated using a single complex-valued constant.

Note that the normalized iSCAT signal is dependent on the square root of the background signal on the background signal (Eq. (5)). Since the background signal can vary between measurements, the quantified iSCAT signal is multiplied by the square-root of the median background signal. In particular, the amplitude of the reflections between the media and the glass in the microfluidic channel depends on the refractive index difference between the media and the glass coverslip. Thus, this correction is needed to maintain a stable DAISY radius as the media refractive index changes.

1.8.1 Z-dependence of the optical signals

As the LFAF attenuates plane waves and point particles differently, a potential depth dependence of the attenuation affects the signal quantification for twilight holography. Moreover, although the U-Net is trained not to have a depth dependence, it can still have a depth dependence in the case of experimental data. To investigate this potential effect, the optical signals from the sample of 105 nm radius polystyrene spheres are inspected as a function of the depth position (Figure S6). It is apparent that, in particular, the iSCAT signal estimation has a depth dependence when the average z-position significantly differs from the middle position, mainly when the z-position difference from the center is more significant than 5 microns. To minimize its effect on the DAISY estimation, only particles with an average depth position within 5 µm from the center position are included in the analysis.

1.9 Quantification of the DAISY radius

The DAISY radius (denoted by r_{DAISY}) is defined as the smallest radius of a homogeneous sphere suspended in water displaying the same scattering ratio and polarizability. To relate the scattering ratio to size, the scattering ratio is first associated with the generalized optical form factor, which is related to particle size. The relation between the generalized optical form factor to particle radius requires that both the C and q values in Eq. 4 in the main text are known.

Although twilight holography and iSCAT measures have an angle between the incoming and scattered light of either 0 or 180 degrees, the optical particle signal does not necessarily average such that the mean signal within the collection angles of the objective lens is best described using 0 or 180 degrees angles. For complex-valued optical field measurements the

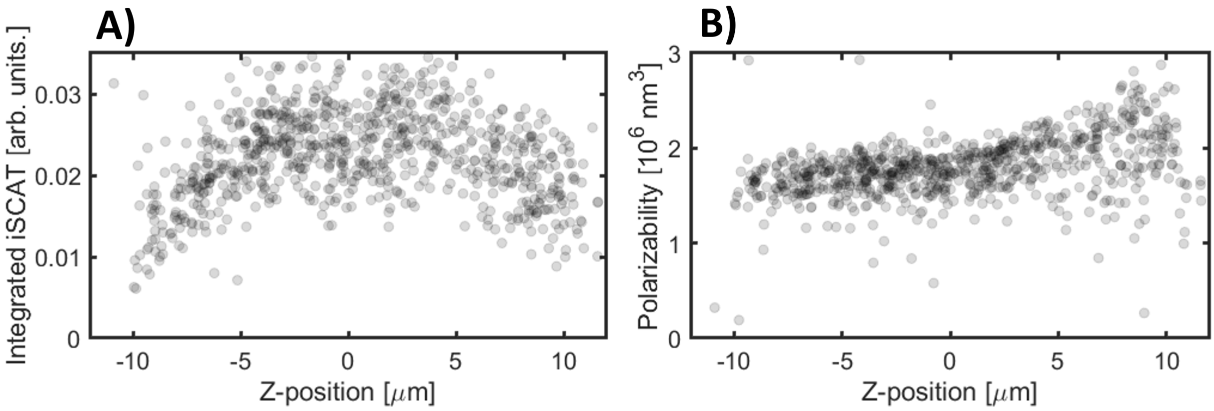

Figure S6: **Z-dependence of the iSCAT and twilight holography signal for 105 nm radius polystyrene suspended in water**. **A** The iSCAT signal and **B** twilight holography signal both display a slight z-dependent particle signal, in particular for when the z-position difference from the center is larger than 5 microns.

range of collection angles does not affect the integrated particle signal [14]. The corresponding mathematical details are presented in the Supplementary Information of Ref. [14], but in short it comes from that spatial integration of $\exp(ik \cdot r)dr$ results in a Dirac delta function for k, which occurs when measuring complex-valued optical fields.

However, the range of collection angles affects the integrated optical signal when only the scattering amplitude is measured, which is the case for iSCAT. Using an oil immersion objective with a numerical aperture of 1.3, the maximum collection angle is 77 degrees for a particle in water. Taking the scattering from a 100 nm radius particle with a refractive index of 1.5, the Mie scattering weighted average angle when considering the difference in amount of scattered light at each angle and the Fresnel equation for transmission, deviates from 180 degrees with 41 degrees. For particle radius around 100 nm, the average angle changes within 1 degree. For this reason, only the iSCAT signal has a form factor different from one, and the scattering angle used in Mie theory to estimate the DAISY radius is 140 degrees.

With q and the refractive indices set, C is quantified by starting each measurement series by measuring the 85 nm and 105 nm radius polystyrene particles in water, where C is estimated by minimizing the difference to Mie theory reference curve using lsqcurvefit in Matlab assuming a particle refractive index of 1.58.

1.9.1 Comparison in precision of the DAISY radius

Since the DAISY radius is based on estimating the optical signal, it does not rely on statistical averaging like the hydrodynamic radius. Thus, the width of the DAISY radius estimation reflects the true size distribution combined with the uncertainty in the DAISY radius estimate.

To evaluate the spread of the DAISY radius estimate, the DAISY radius, the simultaneously measured hydrodynamic radius, and the hydrodynamic radius from nanoparticle tracking analysis (NanoSight LM10 module, Malvern Instruments Ltd., United Kingdom; 488 nm laser) using a shortest track length of 30 are shown in Figure S7. The widths of the DAISY radius distributions are all smaller or comparable to the width of the obtained hydrodynamic radius, where the width of the DAISY radius distribution is different for different particle samples. For example, for the 150 nm radius silica spheres the DAISY radius cannot take value over 170 nm, which limits the potential spread. For this reason, the uncertainty in the DAISY radius estimation depends on both the particles optical signals and size. However, the uncertainty of the

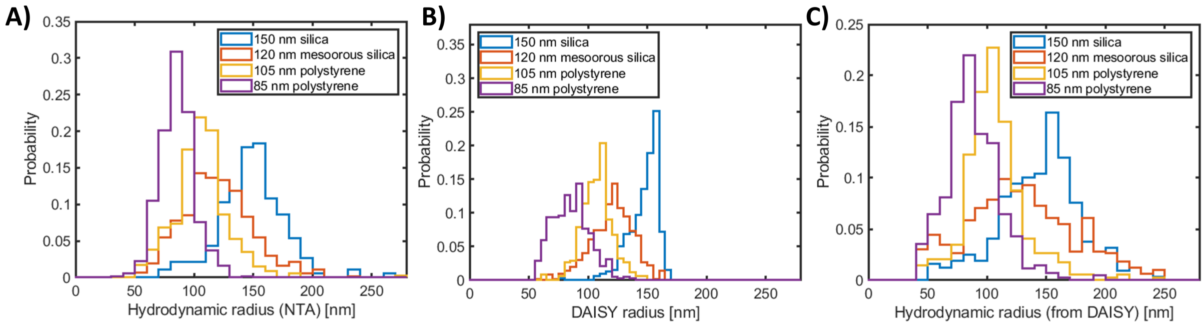

Figure S7: **Comparison of the obtained size distributions from nanoparticle tracking analysis (NTA) and DAISY radius**. Particle size estimates for two polystyrene samples, one silica sample, and one mesoporous silica sample measured using **A** the hydrodynamic radius measured in NTA, **B** the DAISY radius and **C** the simultaneously obtained hydrodynamic radius from the DAISY measurement.

DAISY radius estimate is never the less comparable or better than that of the hydrodynamic for a minimum track length of 30.

1.9.2 The single-valued relation to DAISY radius

When estimating the DAISY radius, both the single particle scattering ratio and polarizability are used. The inclusion of particle polarizability when relating the scattering ratio to size is to include the weak particle refractive index dependence during the particle sizing (Figure 1E in the main text). Specifically, for a given particle refractive index, the relation between scattering ratio and size is unique when the radius is less than $\sim$ 170 nm. When the particle refractive index is unknown, for any measured scattering ratio, there is a range of particle sizes and refractive indices it theoretically could correspond to (Figure 1E in the main text).

When calculating the polarizability for the different particle refractive indices and sizes corresponding to a certain scattering ratio, they all correspond to different polarizability values (Figure S8). Thus, the DAISY radius is uniquely defined using the experimentally available scattering ratio and polarizability. This, in turn, reduces the uncertainty in the size estimation for particles beyond the weakly scattering limit, which is why both the scattering ratio and polarizability is used when estimating the DAISY radius.

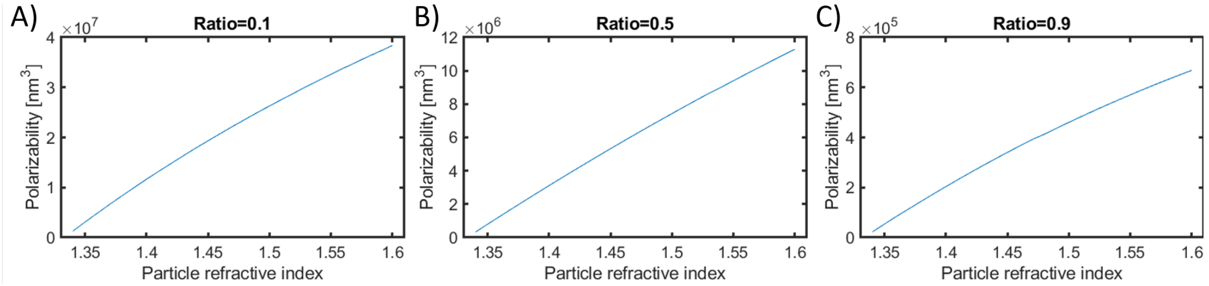

Figure S8: **The relation between scattering ratio and polarizability**. For a given scattering ratio, **A**=0.1, **B**=0.5, and **C**=0.9, all the different particle sizes and refractive indices theoretically could correspond to different polarizability values. Thus, the DAISY radius is uniquely defined when considering both the scattering ratio and polarizability.

2 Relating the optical signal to particle properties

Eq. 1 in the Main text is based on the relation between particle size and the differential scatter cross section, where the scattering cross section is the angular integral of the differential scattering cross section [15]. In the case of using Rayleigh-Debye-Gans theory (RDG), the differential scatter cross section for linearly polarized light at a certain angle is related to the optical form factor by [15, 16]

$$\sigma_{dif} \propto |\alpha|^2 \cos^2(\theta)|f(q_f, \rho)|^2 \tag{3}$$

where α is the particle polarizability and θ is the angle between the scattering direction and the propagation direction of the incoming light [15]. Note that since the same range of scattering angles is measured in both iSCAT and twilight holography, the α and the $\cos(\theta)$ factors are canceled out when taking the ratio between the two signals. Thus, by relating the particle signals data to the forward and backscattering cross sections, their ratio is related to the optical form factor, which can be used to estimate particle size.

2.1 Generalization of the optical form factor using Mie

Since Mie theory is an exact optical scattering theory for homogeneous spheres, it can be used to analyze the optical scattering even when the assumptions behind RDG are no longer valid.

However, when using Mie theory the particle refractive index needs to be assumed when relating the optical scattering ratio to particle size.

For this reason, the DAISY radius is defined as the smallest radius of a homogeneous sphere suspended in water displaying the same scattering ratio and polarizability, where the polarizability information includes the effect of particle refractive index during the size estimate. Using the scattering ratio makes it similar to the optical form factor, which is why the term "generalized form factor" is used, where the generalized form factor, in turn is related to particle size using Mie calculations. The Mie calculations were done using Matscat [17] for which the particle scattering signals were calculated using either 532 nm or 660 nm light. The scattering ratio was normalized to be one when the particle radius approached zero. The media refractive index is 1.335, and the scattering angles are 41 degrees (Section 2.5).

2.2 iSCAT particle signal

The intensity detected in the iSCAT setup results from the interference between the reflected field with intensity $|E_{ref}|^2 = (2r)^2|E_{inc}|^2$, where $2r$ comes from the two reflections in the microfluidic channel and the scattered field from the particle with intensity $|E_{scat}|^2 = |s|^2|E_{inc}|^2$. This means that the measured iSCAT image can be described as [4]

$$I_{\text{iSCAT}} \propto 4|E_{inc}|^2(r^2 + r|s|\cos(\phi) + 0.25 \times |s|^2). \tag{4}$$

where ϕ is the phase difference between the two optical fields. When normalizing the image using the background intensity $|E_{ref}|^2$, the iSCAT contrast is [4]

$$C = 0.25 \times \left(\frac{|s|}{r}\right)^2 + 2\frac{|s|}{r} = 0.25 \times \beta^2\sigma_{back} + \beta\sqrt{\sigma_{back}} \tag{5}$$

where σ_{back} is the particle backscattering cross-section, and β is a proportionality constant of the ratio between $|s|$ and and $\sqrt{\sigma_{back}}$ as well as $1/r$. Note that since $\sigma_{dif} \propto |\alpha|^2\cos^2(\theta)|f(q_f,\rho)|^2$, relating σ_{back} to $|\alpha|$ requires complementary size information when $|f(q_f,\rho)|^2$ no longer can be approximated with 1, where the value of $|f(q_f,\rho)|$ for different particle sizes can be seen in Figure 1E in the main text.

For smaller particles the $\beta^2\sigma_{scat}$ is typically neglected [18]. For larger particles, both terms need to be considered when relating the iSCAT signal to particle properties. To understand which regime DAISY operates in, we note that a typical value for $\beta = 0.03/\text{nm}$ [4]. Moreover, a typical forward scattering cross section value for the used particles is around $\sigma_{forw} \approx 10^3$ nm^2. As the optical form factor for particles with a radius of 100 nm is ~ 0.5, the backscattering cross section is $\sigma_{back} = \sigma_{forw}|f|^2 \approx 0.25 \times 10^3$ nm^2. This makes that

$$\frac{4\beta\sqrt{\sigma_{back}}}{\beta^2\sigma_{back}} \approx 8 \tag{6}$$

Thus, the term proportional to the optical field dominates the particle intensity term by almost an order of magnitude.

To minimize the effect of the $\beta^2\sigma_{back}$ term in the size estimation, inspired by previous solutions in optical coherent imaging [19], we used a U-Net that is trained only to estimate $2\beta\sqrt{\sigma_{back}}$. This minimizes the bias when neglecting the $\beta^2\sigma_{back}$ term. Details about the training of the U-Net are discussed in Section 1.6.1.

2.3 Twilight holography particle signal

The relation between the optical field and the particle polarizability α is shown in Ref [14]. Using their end-result [14],

$$\alpha = \frac{i\lambda_0}{n_m \pi} \iint \mathrm{dxdy}\Big(1 - \bar{t}(x,y)\Big), \tag{7}$$

where $\bar{t}(x,y)$ is the field transmittance normalized to the background signal and n_m is the refractive index of the surrounding media. In twilight holography, the optical field from a particle is quantified relative to the background signal. For this reason, $1 - \bar{t} = -E_p$. The integrated optical field is therefore

$$\iint \mathrm{dxdy} E_{part}(x,y) = -\iint \mathrm{dxdy}\Big(1 - \bar{t}(x,y)\Big) = \frac{i n_m \pi}{\lambda_0}\alpha. \tag{8}$$

From this relation, the integrated imaginary part of the optical field is related to the real part of the polarizability, which is Eq. 2 in the Main text. Moreover, the squared modulus of the polarizability is proportional to the scattering cross section [20]. Thus, the absolute value of the integrated optical fields is proportional to the square root of forward scattering cross section, which is used when deriving the relation between scattering ratio and particle properties.

2.4 Media refractive index dependence in particle scattering from Mie theory

If the refractive index of the surrounding media is slightly underestimated, then relating the scattering to particle radius, as if the refractive index is slightly higher than water when estimating the DAISY radius, the size estimate will be slightly affected. The sensitivity of the different parameters used when performing the Mie calculations is therefore analyzed to estimate this effect.

In Mie theory, the scattering cross section of a sphere is expressed as an infinite sum [15]. Each term depends on two parameters that depend on the particle and the surrounding media. Following the notation of Bohren and Hoffman [15], these two parameters are the relative refractive index $m = n_p/n_m$ and the size parameter x,

$$x = \frac{2\pi n_m R}{\lambda_0}, \quad m = \frac{n_p}{n_m}, \tag{9}$$

where n_m and n_p are the refractive indices of the media and the particle, respectively. These two parameters enter the Mie coefficients both as a product and separate factors.

The product $xm = \frac{2\pi n_p R}{\lambda_0}$ has no media refractive index dependence. The particle radius R in x will be overestimated when n_m is underestimated. Focusing on m, we can write $n_p = n_m^0 + dn$, where a deviation in media refractive index changes $n_m = n_m^0 + \delta n$. This affects m as

$$m = \frac{n_m^0 + dn}{n_m^0 + \delta n} = \frac{1 + dn/n_m^0}{1 + \delta n/n_m^0} \approx 1 + (dn - \delta n)/n_m^0. \tag{10}$$

$(dn - \delta n)/n_m^0$ is proportional to the particle polarizability when the particle refractive index does not deviate too much form that of the media (Main Text). Thus, including the estimated particle polarizability in the DAISY radius gives a first approximation the correct m even when the refractive index of the surrounding media is not precisely known.

Following the media refractive index dependence on the different factors, when estimating the DAISY radius the main influence of imprecise information about the refractive index of the surrounding media is that it affects x. An unaccounted change in media refractive index from 1.335 to 1.37 affects the particle radius in x with 2.6%. Given that the results from Mie calculations should also be consistent with RDG theory in the small weakly scattering limit, an unaccounted change in media refractive index from 1.335 to 1.37 results in a likely overestimation in DAISY radius with at most 2.6%.

2.5 Optical form factor of fractal aggregates

RDG theory is valid for particles that fulfill $|n_p/n_m - 1| \ll 1$ and $|n_p/n_m - 1|kR \ll 1$, where $k = \frac{2\pi n_m}{\lambda}$ [15]. Since particle aggregates have a refractive index close to that of the surrounding media, their optical signal ratio can be analyzed using RDG theory instead of Mie theory.

The form factor of isotropic objects under the RDG approximation is given by

$$f(q) = 4\pi V^{-1} \int dr r^2 \frac{\sin qr}{qr} \rho(r), \tag{11}$$

where $\rho(r)$ is the local density of scatterers, $q = 2k \sin\theta/2$ and θ the scattering angle.

For a fractal aggregate the particle density decays as $\rho(r) \sim r^{D_f - 3}$ as a function of the radial distance r. Instead of deriving the optical form factor for the optical field, we will here derive the optical structure factor for the scattering intensity $S(q, R)$, where $|f(q, R)| = \sqrt{S(q, R)}$. The general expression for the structure factor is [21]

$$S(\vec{q}) = \iint dr_i dr_j \; \rho(\vec{r}_i)\rho(\vec{r}_j) \exp\Big(i\vec{q}\cdot(\vec{r}_i - \vec{r}_j)\Big). \tag{12}$$

By introducing the self-convolution density function

$$g(\vec{r}) = \int dr_i \; \rho(\vec{r} - \vec{r}_i)\rho(\vec{r}_i) \tag{13}$$

it is possible to rewrite Eq. (12) under the assumption of isotropy ($S(\vec{q}) = S(q)$ and $g(\vec{r}) = g(r)$) as [21]

$$S(q) = 4\pi \int dr \; g(r) r^2 \frac{\sin qr}{qr}. \tag{14}$$

For fractal aggregates the general expected form of the self-convolution density function is [21]

$$g(r) = A r^{D_f - d} \exp\Big(-(r/\xi)^\beta\Big) \tag{15}$$

where A is a constant, d is the spatial dimension, D_f is the fractal dimension, ξ is a characteristic length and β is the cut-off exponent. A typical value for β is 2, in which $\xi = \sqrt{\frac{4\times 3}{5D_f}}$ [21]. Using these values, the expression for the structure factor that is used in this work is

$$S(q, R) = 4\pi A \int dr \; r^{Df-1} \exp\Big(-(r^2/R^2)\frac{5D_f}{4\times 3}\Big) \frac{\sin qr}{qr}, \tag{16}$$

where A is chosen such that $S(q, R = 0) = 1$. This expression is solved numerically using numerical integration in MATLAB.

When estimating the DAISY radius of the fractal aggregates in the Main text, the ratio from the optical form factor is related to DAISY radius having the same scattering ratio. The hydrodynamic radius of the aggregates is assumed to be the same as R in the form factor derivation.

2.6 DAISY for larger particles

Depending on particle type, the relation between scattering ratio and particle size can become multi-valued for larger particles. This aspect of DAISY affects its ability to distinguish different particle types from each other in the larger particle limit.

Theoretical DAISY radius and hydrodynamic radius curves for homogeneous spheres and fractal aggregates are displayed in Figure S9, where the size range goes beyond the unique signal scaling for homogeneous spheres. The scattering ratio from homogeneous spheres with a radius larger than 170 nm gradually becomes similar to that of a fractal aggregate. However, for particle radius smaller than 400 nm their apparent fractal dimension is larger than 2.7, which is significantly higher than that one expects for typical particle aggregates (between 1.5 and 2.3 [22–24]). For this reason, particle detections that are centered around a fractal dimension larger than 2.7 is an indication of non-aggregates. Similarly, particle detections centered around a fractal dimension less than 2.7 indicate the presence of aggregates (or optically similar) structures.

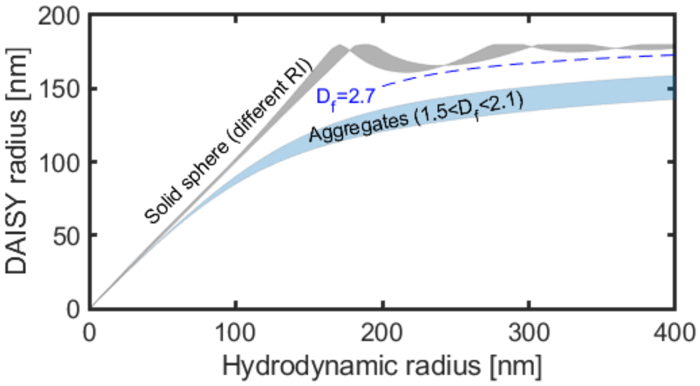

Figure S9: **DAISY for particle sizes outside the region of unique relation between scattering ratio and size**. Homogeneous spheres up to a radius of around 170 nm have a unique signal ratio-size relation, whereas aggregates always have a unique signal ratio-size relation. For spheres larger than 170 nm their signal ratio-size value could be described as a fractal aggregate with a fractal dimension that is larger than 2.7 .

2.7 Practical lower size limit for DAISY

Following Figure 1E in the main text, a unique relation exists between the optical form factor and size for arbitrarily small particles. However, the estimated size will be increasingly sensitive to small errors in the estimated form factor as the particle size decreases toward the Rayleigh limit. Therefore, in practice, the smallest quantifiable size can be estimated based on the noise level of the experimental system.

Assuming that the backward and forward scattered amplitudes E_{b} and E_{f} are quantified from individual scattering patterns with an uncertainty σ_{b} and σ_{rmf}, respectively, the estimated form factor of an individual particle f is related to its true form factor $\tilde{f}$ as

$$f(q;R) = \tilde{f}(q;R) + \tilde{\sigma}/\sqrt{N}\epsilon, \tag{17}$$

where $\tilde{\sigma} = \sqrt{\sigma_\mathrm{b}^2/E_\mathrm{b}^2 + \sigma_\mathrm{f}^2/E_\mathrm{f}^2}$, N is the number of time points in the particle trace, and ϵ is a normal-distributed random number. For small particles, the form factor in the backward direction is related to particle size as $f(2k;R) \approx 1-(2/5)(kR)^2$ [25], indicating that the estimated size R is related to the true size $\tilde{R}$ as

$$\left(R/\tilde{R}\right)^2 - 1 = (5/2)\tilde{\sigma}/\sqrt{N}\epsilon\left(k\tilde{R}\right)^{-2}. \tag{18}$$

Now, $\tilde{\sigma}$ estimates the relative error in signal quantification of individual scattering patterns and is inversely proportional to the scattering amplitudes, which are proportional to particle polarizability for small particles. Therefore, $\tilde{\sigma} \approx \sigma_0(4\pi/3\Delta n\tilde{R}^3)^{-1}$, which allows Eq. (18) to be rewritten as

$$\left(R/\tilde{R}\right)^2 - 1 = (5/2)(4\pi/3\Delta n)^{-1}\sigma_0/\sqrt{N}\epsilon\left(k^2\tilde{R}^5\right)^{-1}. \tag{19}$$

When accurate particle sizing occurs, it can be assumed that difference between the estimated particle radius and true particle radius is small enough such that $|(R/\tilde{R})^2 - 1| < 1$. Since the same should be true for the right-hand side of the equation, we finally have a lower size limit as

$$\tilde{R}^5 > (5/2)(4\pi/3\Delta n)^{-1}\sigma_0/\sqrt{N}k^{-2} \tag{20}$$

Thus, the lower size limit is set by the noise level in the system σ_0, the track length N, and the illumination wavelength through $k = (2\pi)/\lambda$. To estimate the parameter σ_0, we note that the lowest polarizability that is detectable in a single frame amounts to $\sim 5.3 \times 10^{-4}\mathrm{\mu m}^3$, corresponding to the polarizability of a 75 nm radius silica particle. Adopting this value for σ_0, one finds that a 70 nm polystyrene particle can be sized based on the scattering pattern from a single observation. Averaging the scattering patterns over several frames enables accurate quantification of smaller particles. For example, averaging the scattering pattern over 1000 frames leads to the lowest quantifiable size, 35 nm for polystyrene particles and 42 nm for silica. Moreover, this lower practical limit can also be improved by improving the signal to noise ratio in the measured images. Thus, the future improvements of the microscopy setup will likely improve this lower practical limit.

References

[1] E. Olsén, B. Midtvedt, A. González, F. Eklund, K. Ranoszek-Soliwoda, J. Grobelny, G. Volpe, M. Krzyzowska, F. Höök, and D. Midtvedt, "Label-free optical quantification of material composition of suspended virus-gold nanoparticle complexes," *arXiv preprint arXiv:2304.07636, submitted on 15 Apr 2023 (accessed 2023-08-10),* ***https://arxiv.org/abs/2304.07636***, 2023.

[2] K. Goto and Y. Hayasaki, "Three-dimensional motion detection of a 20-nm gold nanoparticle using twilight-field digital holography with coherence regulation," *Optics letters*, vol. 40, no. 14, pp. 3344–3347, 2015.

[3] D. Cole, G. Young, A. Weigel, A. Sebesta, and P. Kukura, "Label-free single-molecule imaging with numerical-aperture-shaped interferometric scattering microscopy," *ACS photonics*, vol. 4, no. 2, pp. 211–216, 2017.

[4] A. D. Kashkanova, M. Blessing, A. Gemeinhardt, D. Soulat, and V. Sandoghdar, "Precision size and refractive index analysis of weakly scattering nanoparticles in polydispersions," *Nature methods*, vol. 19, no. 5, pp. 586–593, 2022.

[5] D. Lee, M. Lee, H. Kwak, Y. S. Kim, J. Shim, J. H. Jung, W.-s. Park, J.-H. Park, S. Lee, and Y. Park, "High-fidelity optical diffraction tomography of live organisms using iodixanol refractive index matching," *Biomedical Optics Express*, vol. 13, no. 12, pp. 6404–6415, 2022.

[6] M. K. Kim, "Principles and techniques of digital holographic microscopy," *SPIE Reviews*, vol. 1, no. 1, p. 018005, 2010.

[7] O. Ronneberger, P. Fischer, and T. Brox, "U-net: Convolutional networks for biomedical image segmentation," *CoRR*, vol. abs/1505.04597, 2015.

[8] B. Midtvedt, S. Helgadottir, A. Argun, J. Pineda, D. Midtvedt, and G. Volpe, "Quantitative digital microscopy with deep learning," *Applied Physics Reviews*, vol. 8, no. 1, p. 011310, 2021.

[9] D. P. Kingma and J. Ba, "Adam: A method for stochastic optimization," *arXiv preprint arXiv:1412.6980*, 2014.

[10] M. Abadi, A. Agarwal, P. Barham, E. Brevdo, Z. Chen, C. Citro, G. S. Corrado, A. Davis, J. Dean, M. Devin, S. Ghemawat, I. Goodfellow, A. Harp, G. Irving, M. Isard, Y. Jia, R. Jozefowicz, L. Kaiser, M. Kudlur, J. Levenberg, D. Mané, R. Monga, S. Moore, D. Murray, C. Olah, M. Schuster, J. Shlens, B. Steiner, I. Sutskever, K. Talwar, P. Tucker, V. Vanhoucke, V. Vasudevan, F. Viégas, O. Vinyals, P. Warden, M. Wattenberg, M. Wicke, Y. Yu, and X. Zheng, "TensorFlow: Large-scale machine learning on heterogeneous systems," 2015. Software available from tensorflow.org.

[11] R. Parthasarathy, "Rapid, accurate particle tracking by calculation of radial symmetry centers," *Nature methods*, vol. 9, no. 7, pp. 724–726, 2012.

[12] D. Midtvedt, F. Eklund, E. Olsén, B. Midtvedt, J. Swenson, and F. Höök, "Size and refractive index determination of subwavelength particles and air bubbles by holographic nanoparticle tracking analysis," *Analytical Chemistry*, vol. 92, no. 2, pp. 1908–1915, 2019.

[13] C. L. Vestergaard, P. C. Blainey, and H. Flyvbjerg, "Optimal estimation of diffusion coefficients from single-particle trajectories," *Physical Review E*, vol. 89, no. 2, p. 022726, 2014.

[14] S. Khadir, D. Andrén, P. C. Chaumet, S. Monneret, N. Bonod, M. Käll, A. Sentenac, and G. Baffou, "Full optical characterization of single nanoparticles using quantitative phase imaging," *Optica*, vol. 7, no. 3, pp. 243–248, 2020.

[15] C. F. Bohren and D. R. Huffman, *Absorption and scattering of light by small particles.* John Wiley & Sons, 2008.

[16] P. W. Barber and D.-S. Wang, "Rayleigh-gans-debye applicability to scattering by nonspherical particles," *Applied optics*, vol. 17, no. 5, pp. 797–803, 1978.

[17] J.-P. Schäfer, *Implementierung und Anwendung analytischer und numerischer Verfahren zur Lösung der Maxwellgleichungen für die Untersuchung der Lichtausbreitung in biologischem Gewebe.* PhD thesis, Universität Ulm, 2011.

[18] L. Priest, J. S. Peters, and P. Kukura, "Scattering-based light microscopy: From metal nanoparticles to single proteins," *Chemical Reviews*, vol. 121, no. 19, pp. 11937–11970, 2021.

[19] Y. Rivenson, Y. Wu, and A. Ozcan, "Deep learning in holography and coherent imaging," *Light: Science & Applications*, vol. 8, no. 1, p. 85, 2019.

[20] S. A. Maier, *Plasmonics: fundamentals and applications.* Springer Science & Business Media, 2007.

[21] C. Sorensen, "Light scattering by fractal aggregates: a review," *Aerosol Science & Technology*, vol. 35, no. 2, pp. 648–687, 2001.

[22] D. W. Schaefer, J. E. Martin, P. Wiltzius, and D. S. Cannell, "Fractal geometry of colloidal aggregates," *Physical Review Letters*, vol. 52, no. 26, pp. 2371–2374, 1984.

[23] M. Carpineti, F. Ferri, M. Giglio, E. Paganini, and U. Perini, "Salt-induced fast aggregation of polystyrene latex," *Physical Review A*, vol. 42, no. 12, p. 7347, 1990.

[24] B. Midtvedt, E. Olsén, F. Eklund, F. Höök, C. B. Adiels, G. Volpe, and D. Midtvedt, "Fast and accurate nanoparticle characterization using deep-learning-enhanced off-axis holography," *ACS Nano*, vol. 15, no. 2, pp. 2240–2250, 2021.

[25] P. J. Wyatt, "Light scattering and the absolute characterization of macromolecules," *Analytica chimica acta*, vol. 272, no. 1, pp. 1–40, 1993.
